# Chitosan-Zinc-Ligated Hydroxychloroquine: Molecular Docking, Synthesis, Characterization, and Trypanocidal Activity against *Trypanosoma evansi*

**DOI:** 10.3390/polym16192777

**Published:** 2024-09-30

**Authors:** Anju Manuja, Ruma Rani, Nisha Devi, Monika Sihag, Swati Rani, Minakshi Prasad, Rajender Kumar, Tarun Kumar Bhattacharya, Balvinder Kumar

**Affiliations:** 1 ICAR-National Research Centre on Equines, Hisar 125001, India; rumasaharan@gmail.com (R.R.); nishu.sharma57605@gmail.com (N.D.); monasihag02@gmail.com (M.S.); spahwa999@gmail.com (S.R.); minakshi.abt@gmail.com (M.P.); rkg.nrce@gmail.com (R.K.); bhattacharyatk@gmail.com (T.K.B.); 2 Department of Chemistry, Chaudhary Bansi Lal University, Bhiwani 127021, India

**Keywords:** chitosan, zinc, hydroxychloroquine, molecular docking, trypanocidal activity

## Abstract

The existing treatments against *Trypanosoma evansi* are faced with several drawbacks, such as limited drug options, resistance, the relapse of infection, toxicity, etc., which emphasizes the necessity for new alternatives. We synthesized novel metal-based antiparasitic compounds using chitosan, hydroxychloroquine (HC), and ZnO nanoparticles (NPs) and characterized them for size, morphology, chemical interactions, etc. Molecular docking and protein interaction studies were performed in silico to investigate the inhibitory effects of HC, zinc-ligated hydroxychloroquine (HCZnONPs), and chitosan-zinc-ligated hydroxychloroquine (CsHCZnONPs) for two key proteins, i.e., heat shock protein 90 (Hsp90) and trypanothione reductase associated with *T. evansi*. In vitro trypanocidal activity and the uptake of zinc ions by *T. evansi* parasites were observed. The formulation was successfully synthesized, as indicated by its size, stability, morphology, elemental analysis, and functional groups. CsHCZnO nanoparticles strongly inhibit both Hsp90 and trypanothione reductase proteins. The inhibition of Hsp90 by these nanoparticles is even stronger than that of trypanothione reductase when compared to HC and HCZnONPs. This suggests that the presence of polymer chitosan enhances the nanoparticles’ effectiveness against the parasite. For the first time, CsHCZnO nanoparticles exhibited trypanocidal activity against *T. evansi*, with complete growth inhibition being observed at various concentrations after 72 h of treatment. Fluorescent microscopy using FluoZin-3 on *T. evansi* culture confirmed the presence of zinc on the surface of parasites. This innovative approach has shown promising results in the quest to develop improved antiparasitic compounds against *T. evansi* with enhanced effectiveness and safety, highlighting their potential as therapeutic agents against trypanosomiasis.

## 1. Introduction

Trypanosomosis, also known as Surra, is a significant and economically important disease caused by the protozoan parasite *Trypanosoma evansi* [1]. This parasite primarily resides within the blood, plasma, tissues, and body fluids of *T. evansi*-infected animals. The disease has a global presence and is particularly impactful in regions like Asia, Africa, and South America. It poses a considerable challenge to livestock productivity in these areas. *T. evansi* has a broad host range, infecting a variety of domestic animals as well as wildlife [2]. Infection leads to various symptoms, including fever, anemia, weakness, weight loss, and sometimes death [3,4]. The parasites can be transmitted by various means, including through biting flies (mechanical transmission), contaminated needles, or vertical transmission from mother to offspring [1].

While trypanosomosis predominantly affects animals, there have been occasional reports of the parasite’s ability to infect humans [5]. Efforts to control and manage trypanosomosis include various strategies, such as using insecticides to control the vectors, administering drugs to infected animals, and implementing measures to reduce animal movement and potential exposure to infected vectors. Limited options are available for treating trypanosomiasis, and many existing drugs, such as quinapyramine sulfate, isometamidium, and diminazene, aceturate exhibit subpar safety, efficacy, and pharmacokinetic profiles [2,6]. There is a critical need to develop medications with better pharmacological activity, bioavailability, and safety to effectively combat this disease [7].

Medicinal inorganic chemistry has shown potential in developing compounds that affect trypanosomatid parasites. Research has led to the creation of various coordination compounds and organometallics that exhibit activity against these parasites [8,9].

Our research group has focused on creating novel metal-based antiparasitic compounds with improved biological characteristics. This involves designing multifunctional metal compounds by combining distinct pharmacophoric moieties to create hybrid molecules [10,11]. These compounds, which include a bioactive metal center and a bioactive ligand, often exhibit dual or multiple modes of action, reduced toxicity, and better selectivity.

Chloroquine and its derivative, hydroxychloroquine (HC), are well known for their effectiveness in treating malaria and autoimmune diseases. They also demonstrated its trypanocidal effect against *Trypanosoma brucei* [12] and its suppressive effect on the parasite in rats [13]. Chloroquine phosphate serves as a zinc ionophore [14]. However, prolonged use of these drugs can lead to notable side effects. Hydroxychloroquine (HC), a less hazardous form, can inhibit lysosomotropic autophagy [15] and has been reported as anti-viral [15,16,17], but it still has adverse effects, including gastrointestinal discomfort, hepatotoxicity, and impaired vision. Zinc is an important mineral that is required for many different body processes. It is well renowned for strengthening the immune system and warding off diseases. Zinc hinders the ability of parasites to proliferate within the body by interfering with the enzymes and proteins necessary for their survival.

We developed a formulation using chitosan, zinc oxide, and hydroxychloroquine to deliver Zn and HC effectively. We utilized chitosan as an effective carrier for zinc and chloroquine to mitigate their adverse effects. Nanocarriers help prevent any harmful impacts of these drugs or chemicals on the host. Chitosan, a biocompatible and biodegradable polysaccharide, was chosen due to its ability to bind easily to the drug. Additionally, these compounds tend to show reduced toxicity and improved selectivity. This formulation has shown inhibitory effects against *T. evansi* proteins, especially Hsp 90 and trypanothione reductase. For the first time, we investigated the inhibitory activity of this complex against *T. evansi*, demonstrating its potential as a safe and effective treatment.

## 2. Materials and Methods

### 2.1. Materials

We acquired chitosan (CS: deacetylated chitin, 75–85% deacetylation) from Sigma-Aldrich Chemicals Private Ltd. (Bangalore, India). The source of zinc sulfate was Qualigens Fine Chemicals Pvt. Ltd. in Mumbai, India. The supplier of HC was Ipca Laboratories (Mumbai, India). Other chemicals and reagents mentioned in this study were also of analytical grade and purchased from Sigma-Aldrich Chemicals Private Ltd. (Bangalore, India). All glassware used in this study was from M/S Borosil (Mumbai, India).

### 2.2. Molecular Docking Studies

Computationally, the binding interaction and energy were evaluated using Autodock software. An in silico analysis of binding interactions and energy was carried out using Autodock software. Three-dimensional structures were prepared with ChemDraw Ultra 15.0 and optimized using Avogadro software 1.2. Torsions and charges were assigned using Autodock 4.2. X-ray structures of Heat shock protein 90 (PDB: 3O6O) and trypanothione reductase protein (PDB: 2WPS) were obtained from the RCSB Protein Data Bank. Protein preparation, along with removal of the B chain and water molecules, the addition of polar hydrogens, and the removal of heteroatoms, was performed using Chimerax 1.8. Subsequently, the protein was processed in Autodock for the addition of Kollman charges, and a pdbqt (Protein Data Bank (PDB), atomic charge (Q) and atom type (T)) file of the protein was saved. A grid map was generated with dimensions of 6.7 × 11.3 × 8.2 Å at the grid center of 0.5 Å. Molecular docking was conducted, and docking results were obtained in dlg format. The pdbqt file for the best conformer was saved using Autodock tools and visualized with Discovery Studio Visualizer.

### 2.3. Synthesis of Chitosan Hydroxychloroquine Zinc Oxide Nanoparticles (CsHCZnONPs)

By utilizing zinc sulfate as a precursor and a microwave-assisted fast approach with slight modifications, as described previously [18], we synthesized dispersible ZnO NP with a morphology resembling flowers. A composite of HC and ZnONP was synthesized in conjugation with chitosan (C), wherein the HC was present in an effective amount of 0.0012–0.012% wt, ZnONP was present in an effective amount of 0.05–0.75% wt, and chitosan polymer was present in an effective amount of 0.01–1% prepared in 0.1% acetic acid. It was filtered through a 0.22 µM membrane and stored at 4 °C until further use.

### 2.4. Characterization of CsHCZnONPs

The synthesized CsHCZnONPs were characterized for various features using different techniques. Dynamic light scattering (DLS) with the Zetasizer nano ZS (Malvern Instruments, Malvern, UK) was used to determine the particle size and surface charge (μ) with PDI @25 °C. Transmission electron microscopy (TEM) was performed by placing a drop on a 400-mesh copper grid covered with carbon, and scanning electron microscopy (SEM) with an EDAX detector was used for morphology and element analysis, respectively. The SEM sample was sputtered with gold, and the measurement was taken at an accelerating voltage of 15 KV. Fourier transform infrared spectroscopy with an IR spectrophotometer (Perkin Elmer Spectrum BX II, Hopkinton, MA, USA) was carried out to observe chemical interactions. For the FTIR analysis, the sample powder was mixed with KBr and a pallet was formed.

### 2.5. In Vitro Propagation of T. evansi

DEAE-cellulose chromatography was utilized to isolate host–cell-free *T. evansi* from mouse blood following the method detailed by Lanham and Godfray in 1970 [19]. The experiment was conducted as per the guidelines of the Institutional Animal Ethics Committee of the ICAR-National Research Center on Equines, Hisar, India (193/GO/Re/SL/99/CPCSEA), approved by NRCE/CPCSEA/2019–20 on 15 February 2020. Mice were housed in an authorized mouse facility at a small animal home in a regulated and pathogen-free environment. Mice were housed on a 12 h light/dark cycle under ambient temperature (22 ± 2 °C) with ad libitum feed and water. Cryostabilized *T. evansi* parasites (T.ev-India-NRCE-Horse1, Hisar, India) were revived in 10% FBS-supplemented HMI-Medium and maintained at 37 °C in a 5% CO_2_ incubator [20].

### 2.6. Efficacy of CsHCZnONPs against T. evansi

Formulated CsHCZnONPs were evaluated for their in vitro growth-inhibitory effects on *T. evansi* parasites (1 × 10^6^/mL). Various concentrations of CsHCZnONPs (250, 125, 62.5, 31.25, 15.62, 7.81, and 3.9 μg/mL) were added to the parasite culture at 24, 48, and 72 h daily. The conventional drug QPS (Quinapyramine sulfate) @10 μg/mL was employed as reference control, whereas DMSO and untreated parasites served as negative controls. The impact of the drug was evaluated by counting the parasite numbers using a Neubauer hemocytometer every 24 h for up to three days. The 50% inhibitory concentration (IC_50_) for the formulation was determined at 24 h, and the IC_50_ values for the nanoformulation were calculated in triplicate using GraphPad prism software version 8.0.2 (263).

The microscopic assessment of parasites treated with different formulations involved preparing slides after 24, 48, and 72 h of treatment and observing them using an upright microscope (Nikon Eclipse E200, Tokyo, Japan) at 1000× magnification.

## 3. Results and Discussion

### 3.1. Molecular Docking

**Figure 1:**
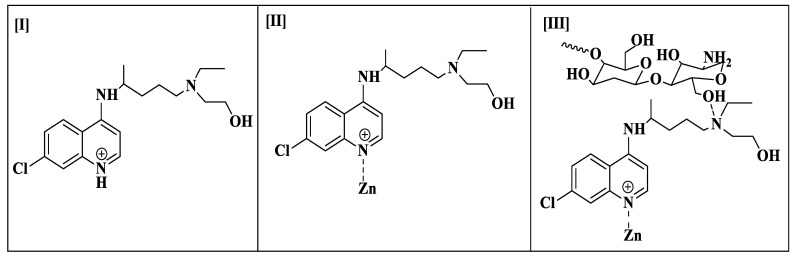
Chemical structures of hydroxychloroquine (**I**), zinc-ligated hydroxychloroquine (**II**), and chitosan-Zn-ligated hydroxychloroquine (**III**).

The molecular chaperone Hsp90 is crucial for the viability and propagation of protozoan parasites as it helps in protein folding and stabilizing key regulatory proteins. *Trypanosoma* species and other protozoan parasites have distinct Hsp90 isoforms not found in the host [21]. This makes it possible to develop drugs that selectively target the Hsp90 of the parasite without interfering with the proteins of the host. Inhibiting Hsp90 in protozoan parasites can lead to misfolded proteins, aggregation, and eventually to parasite death. In order to combat infections, one effective strategy may be to impair the system that controls the quality of proteins. Combining the targeting of Hsp90 with other therapies can enhance the overall therapeutic effect, potentially reducing the development of resistance and improving treatment outcomes [22]. The inhibition of heat shock protein 90 (Hsp90) has been reported as one of the methods that trypanocidal activity against *T. evansi* occurs [23]. Therefore, we performed the docking of HC, HCZnONPs, and CsHCZnONPs on the active site of the Hsp90 protein (PDB: 3O6O) [24] to gain further insights into the binding interaction and orientation of these molecules in the binding pockets. The Hsp90 protein (PDB: 3O6O) has identical bindings of two chains, A and B, and identical binding pockets. The chemical structures of HC, HCZnONP, and CsHCZnONPs are given in Figure 1.

**Figure 2:**
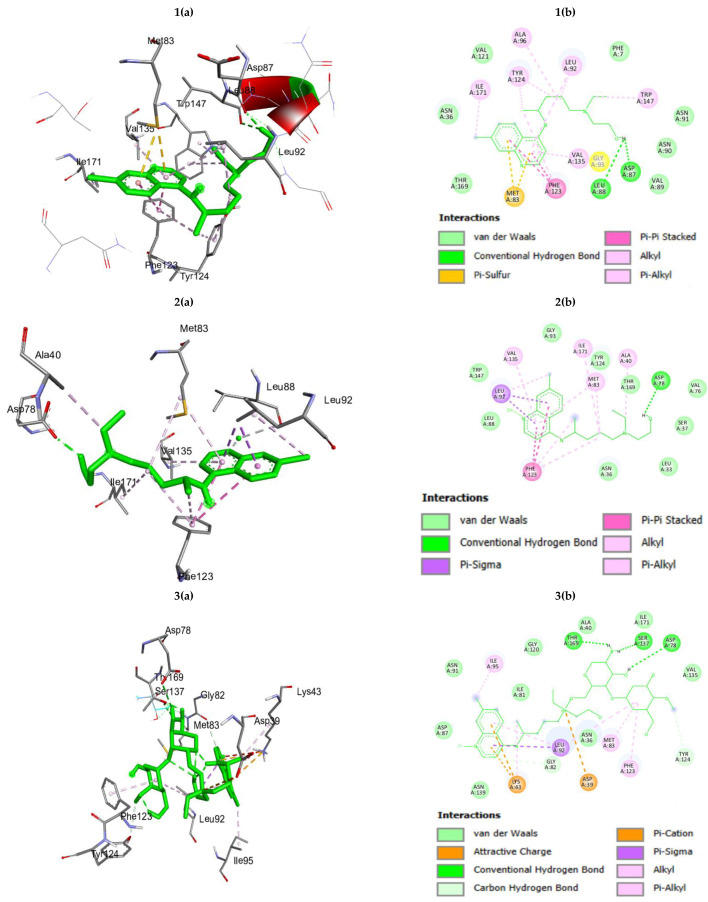
(**a**) Three-dimensional interactions and (**b**) two-dimensional diagram of (**1**) hydroxychloroquine (HC); (**2**) Zn-ligated hydroxychloroquine (HCZnONP); (**3**) chitosan-Zn-ligated hydroxychloroquine (CsHCZnONP) with Hsp 90 protein.

The binding energies of HC, HCZnONP, and CsHCZnONP for the Hsp90 protein were −7.73, −9.88, and −11.01 Kcal/mol, respectively, which suggests that the synthesized formulation (CsHCZnONP) is a good inhibitor of the Hsp90 target protein compared to HC and HCZnONP. The interacting residues for HC, HCZnONP, and CsHCZnONP Hsp 90 are provided in Table 1 and Figure 2.

**Figure 3:**
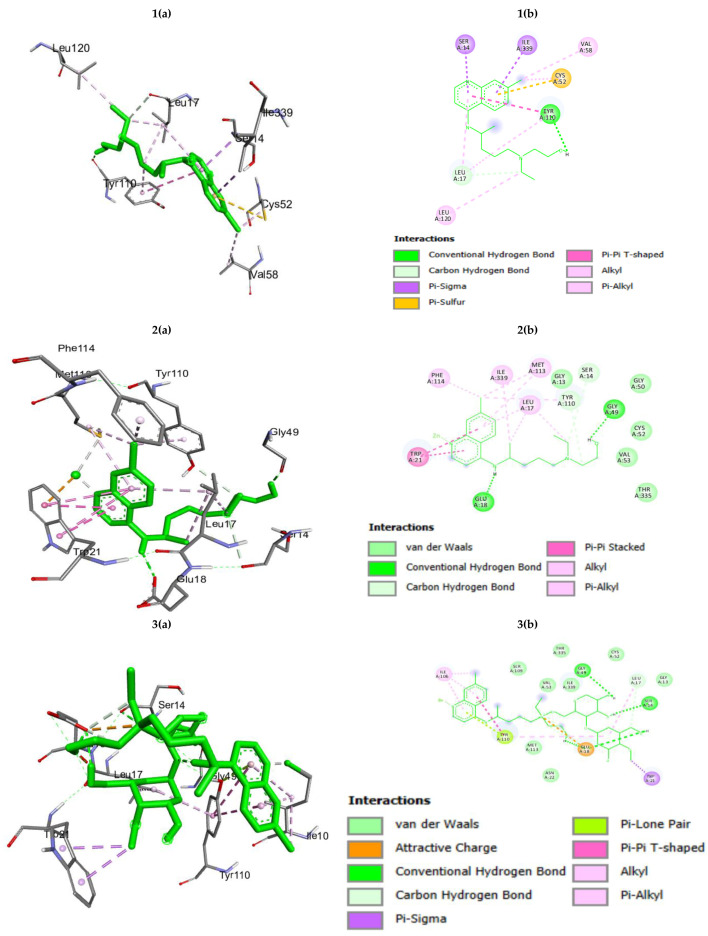
(**a**) Three-dimensional interactions and (**b**) two-dimensional diagram of (**1**) hydroxychloroquine (HC); (**2**) Zn-ligated hydroxychloroquine (HCZnONP); (**3**) chitosan-Zn-ligated hydroxychloroquine (CsHCZnONP) with trypanothione reductase.

The parasite utilizes a redox pathway, which involves the conversion of disulfide trypanothione to dihydro-trypanothione. Therefore, trypanothione reductase, an enzyme of the trypanothione-based metabolic pathway, becomes an important target for the development of trypanocidal drugs [25,26,27]. Its absence in mammalian cells offers a selective pathway for drug development. Therefore, we performed the docking of HC, HCZnONP, and CsHCZnONP on the active site of the trypanothione reductase protein (PDB: 2WPC) [28] to gain further insights into the binding interaction and orientation of these molecules in the binding pocket of NADPH (Table 2 and Figure 3). The binding energy and interacting residues of trypanothione reductase enzyme for HC, HCZnO NPs, and CsHCZnONP were found to be different. The binding energy of trypanothione reductase was −5.96 for HC, −9.00 for HCZnONP, and −9.17 for CsHCZnONP. CsHCZnONP strongly inhibits the target trypanothione reductase enzyme because of its smaller binding energy in comparison to HC and HCZnONP (Table 2).

As a result of molecular docking, it is found that both Hsp 90 and trypanothione reductase are strongly inhibited by synthesized CsHCZnONP, but Hsp90 is a more potent target than trypanothione reductase.

### 3.2. Synthesis and Characterization of CsHCZnO NFs

**Figure 4:**
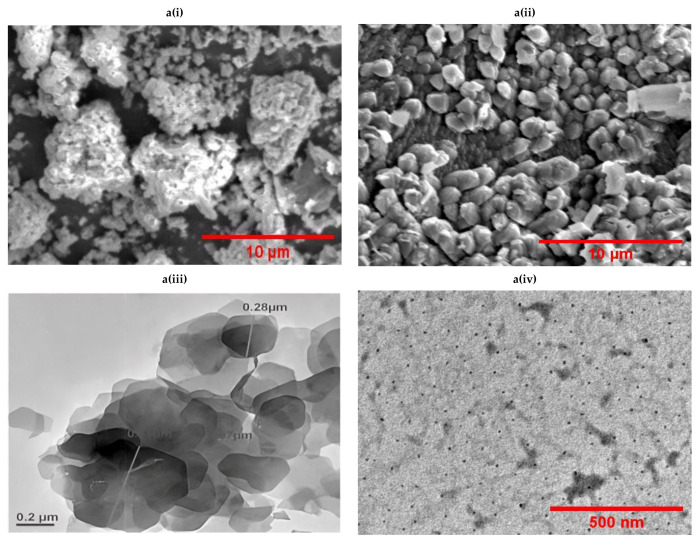

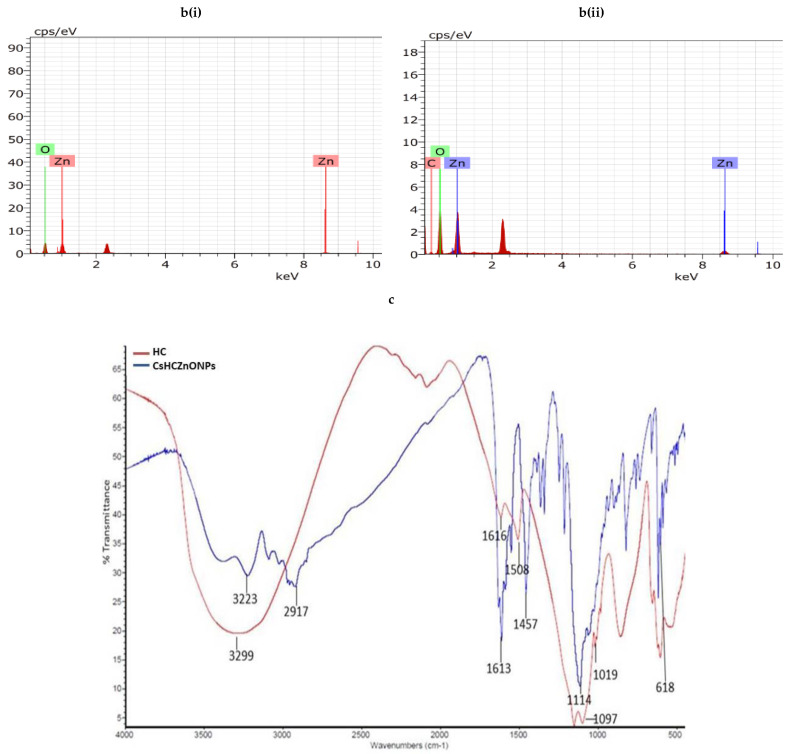
Characterization of chitosan hydroxychloroquine zinc oxide nanoparticles. Images observed under (**a**) scanning electron microscope: (**i**) ZnO NPs, (**ii**) CsHCZnONPs; (**a**) transmission electron microscope: (**iii**) ZnO NPs, (**iv**) CsHCZnONPs. (**b**) Elemental analysis of (**i**) ZnO NPs and (**ii**) CsHCZnO NPs. (**c**) FTIR spectra of HC and CsHCZnO NPs.

The ZnO NPs had an average size of less than 10 nm and a polydispersity index (PDI) of 0.00, suggesting the monodispersive nature of ZnO NPs. In contrast, the CsHCZnO NPs had a viscosity of 0.852, a PDI of 0.282, and a larger size range (252.9 to 1469 nm). This discrepancy in size measurements is attributed to the high swelling capacity of chitosan nanoparticles, causing dynamic light scattering to yield larger hydrodynamic diameters compared to electron microscopy. The SEM analysis revealed that the ZnO NPs exhibited a cauliflower-like morphology, while CsHCZnONPs appeared as individual particles (Figure 4(ai) and Figure 4(aii), respectively). The ZnO NPs were arranged as assorted petals in the shape of a flower-like structure, and CsHCZnONPs appeared as discrete nanoparticles, as determined by TEM (Figure 4(aiii) and Figure 4(aiv), respectively). An elemental analysis also confirmed zinc and oxygen in the ZnO NPs (Figure 4(bi)), and additional carbon along with zinc and oxygen in the CsHCZnONP formulation (Figure 4(bii)), indicating the incorporation of chitosan and HC into the formulation. This analysis reveals distinct differences in their physical and chemical properties, which can be attributed to the presence of chitosan and HC.

The FTIR spectral analysis of HC and CsHCZnONPs reveals the various functional groups present and the shifting of peaks due to the interaction between functional groups of HC and CsHCZnONPs. The spectrum was recorded in the 4000–400 cm^−1^ range. The –OH stretching spectral peak at 3223 cm^−1^ of hydroxychloroquine is shifted to 3299 cm^−1^ in CsHCZnONPs. The aromatic C=C extension in regions 1613 and 1552 cm^−1^ in CHC is shifted to 1616 and 1508 cm^−1^, respectively, in CsHCZnONPs (Figure 4c). The stretching phenomenon from 1114 to 1151 cm^−1^ represents the C-N bending in CsHCZnONPs. Both molecules are present in the formulation, as evident by significant alterations in the FTIR spectra of HC and CsHCZnONPs. The FTIR spectrum indicates significant changes in the functional groups of hydroxychloroquine upon incorporation into the CsHCZnONP formulation. These changes correspond to the chemical interactions between hydroxychloroquine, ZnO NPs, and chitosan, indicating that all components interact within the formulation.

### 3.3. Efficacy of CsHC-ZnONPs against T. evansi

We already reported nanoparticle formulation’s compatibility in Vero cells and SPF embryonated chicken eggs [11]. A cytotoxicity assessment using resazurin dye indicated that ZnO NPs were more toxic than CsHCZnONPs at concentrations exceeding 100 µg/mL. This reduced the cytotoxicity of CsHCZnONPs, which was attributed to the conjugation of HC with ZnO NPs, potentially involving biodegradable materials.

**Figure 5:**
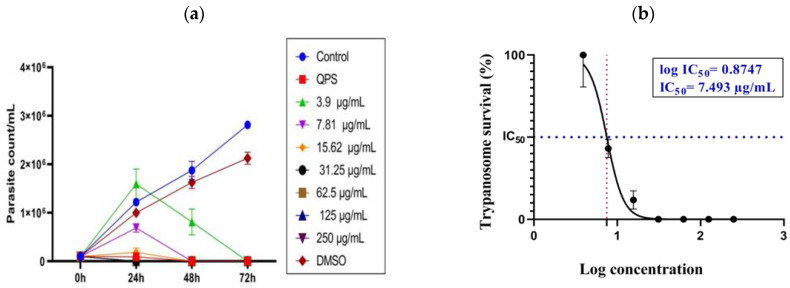
(**a**). Efficacy of chitosan hydroxychloroquine zinc oxide nanoparticles against *Trypanosoma evansi* parasites (standard deviation is shown for triplicate samples). (**b**) IC_50_ and log IC_50_ values of CsHC ZnO NPs.

**Figure 6:**
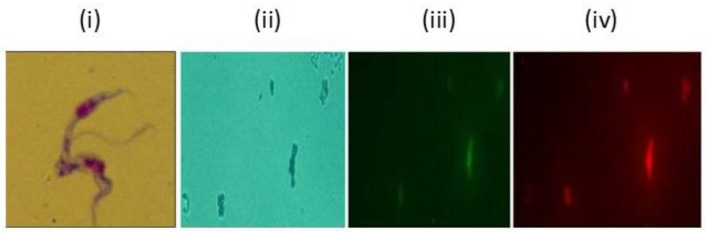
*T. evansi* culture was treated with CsHCZnONPs and stained with FluoZin and lysomotracker. Fluorescent signals were measured by excitation/emission at 490/20 nm and 555/28 nm for Fluozin and lysomotracker respectively. (**i**) Giemsa-stained untreated dividing parasite. (**ii**) *T. evansi* under normal illumination in optical microscope. Same field under fluorescent microscope. (**iii**) Fluozin; (**iv**) Lysomotracker (1000× magnification).

The assessment of the effectiveness of growth inhibition against *T. evansi* showed trypanocidal activity on parasites treated with CsHCZnONPs compared to the negative control. At 24, 48, and 72 h, antagonistic findings were shown in the untreated negative control, indicating a significant increase in the parasite population. The growth inhibitory effects of various formulation concentrations showed varying degrees of significance, which changed as the incubation time increased from 24 h to 72 h. Different treatments at varying concentrations and times (24, 48, and 72 h) are represented in Figure 5a. At 24 h, parasites were completely eliminated at concentrations ranging from 250 to 15.65 μg/mL, and they were reduced at a concentration of 7.81 μg, followed by 3.9 μg/mL. After 48 h, live parasites were not observed even at the 15.62 μg concentration (Figure 5a). After 72 h, complete growth inhibition was observed at all the concentrations in wet smears. In contrast, the untreated *T. evansi* parasites exhibited continuous growth due to dividing parasites. Giemsa-stained parasites revealed dividing parasites in the untreated controls (Figure 6i). Furthermore, QS (10 µg/mL) was employed as a reference control. Compared to the negative control parasite, the QS-treated culture showed a full suppression of the parasites at 24 h. (*p* < 0.0001) The IC_50_ and log IC_50_ values of CsHCZnONPs were 7.493 μg/mL and 0.8747 μg/mL, respectively (Figure 5b).

### 3.4. Intracellular Detection of Zn

Intracellular free zinc ions were also detected using FluoZin-3 probes and a LysoTracker under fluorescent microscopy following the manufacturer’s instructions. FluoZin/LysomoTracker staining demonstrated the presence of ZnO NPs on the surface of the parasites. Giemsa-stained parasites revealed dividing parasites in the untreated control, whereas the CsHCZnONP-treated cultures either showed debris or fragmented trypanosomes. The treated *T. evansi* parasites stained with FluoZin and LysoTracker were observed under an optical microscope (Figure 6ii) and fluorescent microscope with fluorescent signals measured by excitation and emission at 490/20 nm (Figure 6iii) and 555/28 nm (Figure 6iv). Staining with Fluozin revealed the presence of ZnO NPs on the surface of the parasite. Fragmented trypanosomes were also observed, which may have been due to mitochondrial fenestration prior to the complete destruction of the parasite (Figure 6), which probably caused the death of the parasite just like trypanolytic factor Apolipoprotein in human blood forms pores first in endosomal membranes and then in the mitochondrial membranes and plasma membranes [29].

## 4. Conclusions

This study investigated the inhibitory effects of hydroxychloroquine (HC), zinc-ligated hydroxychloroquine (HCZnONPs), and chitosan-zinc-ligated hydroxychloroquine (CsHCZnONPs) on two key proteins, heat shock protein 90 (Hsp90) and trypanothione reductase, associated with *T. evansi*. Molecular docking analyses showed that the CsHCZnO nanoparticles strongly inhibit both Hsp90 and trypanothione reductase proteins. The inhibition of Hsp90 by these nanoparticles is even stronger than that of trypanothione reductase when compared to hydroxychloroquine alone or hydroxychloroquine zinc oxide nanoparticles. This suggests that the presence of chitosan enhances the nanoparticles’ effectiveness against the parasite. A SEM analysis showed a cauliflower-like morphology for the ZnONPs and discrete particles for CsHCZnONPs. An elemental analysis confirmed the presence of zinc and oxygen in the ZnONPs and additional carbon along with zinc and oxygen in CsHCZnONPs. A cytotoxicity assessment in Vero cells and SPF embryonated chicken eggs indicated a reduced toxicity of CsHCZnONPs compared to ZnONPs, which was likely due to the conjugation of hydroxychloroquine with ZnO nanoparticles involving biodegradable polymers.

CsHCZnONPs exhibited trypanocidal activity, with complete growth inhibition being observed at various concentrations after 72 h of treatment. Fluorescent microscopy using FluoZin-3 and LysoTracker probes confirmed the presence of ZnONPs on the surface of the parasites. The treated *T. evansi* culture revealed fragmented trypanosomes, leading to parasite death.

In summary, the outcome of this approach led to the creation of multifunctional compounds that demonstrate dual or multiple modes of action. This study demonstrated the potent inhibitory effects of CsHCZnO nanoparticles against Hsp90 and trypanothione reductase, as well as their trypanocidal activity against *T. evansi*, highlighting their potential as therapeutic agents against trypanosomiasis and for further investigations in animal models. These findings underscore the potential of CsHCZnONPs as a multifaceted therapeutic platform with modified physical and chemical properties that is suitable for various biomedical applications.

## Figures and Tables

**Table 1:** Binding energies and interacting residues with Hsp 90 protein for compounds: hydroxychloroquine (1); Zn-ligated hydroxychloroquine (2); chitosan-Zn-ligated hydroxychloroquine (3).

Sr. No.	Compound	Binding Energy (Kcal/mol)	Interacting Residues
1	Hydroxychloroquine	−7.73	PHE A:7, ASN A:36, MET A:83, ASP A:87, LEU A:88, VAL A:89, ASN A:90, ASN A:91, LEU A:92, GLY A:93, ALA A:96, VAL D:121, PHE A:123, TYR A:124, VAL A:135, TRP A:147, THR A:169, ILE A:171
2	Zn-Ligated Hydroxychloroquine	−9.88	LEU A:33, ASN A:36, SER A:37, ALA A:40, VAL A:76, ASP A:78, MET A:83, LEU A:88, LEU A:92, GLY A:93, PHE A:123, TYR A:124, VAL A:135, TRP A:147, THR A:169, ILE A:171
3	Chitosan-Zn-Ligated Hydroxychloroquine	−11.01	ASN A:36, SER A:37, ASP A:39, ALA A:40, LYS A:43, ASP A:78, ILE A:81, GLY A:82, MET A:83, ASP A:87, ASN A:91, LEU A:92, ILE A:95, GLY A:120, PHE A:123, TYR A:124, VAL A:135, SER A:137, ASN A:139, THR A:169, ILE A:171

**Table 2:** Binding energies and interacting residues with trypanothione reductase for compounds: hyroxychloroquine (1); Zn-ligated hydroxychloroquine (2); chitosan-Zn-ligated hydroxychloroquine (3).

Sr. No.	Compound	Binding Energy (Kcal/mol)	Interacting Residues
1	Hydroxychloroquine	−5.96	SER:14, LEU: 17, CYS:52, VAL:58, TRY:110, LEU:120, ILE:339
2	Zn-Ligated Hydroxychloroquine	−9.00	GLY:13, SER:14, LEU: 17, GLU:18, TRP:21, ASN:22, GLY:49, CYS:52, VAL:53, VAL:58, ILE:106, SER:109, TRY:110, MET:113, LEU:120, THR:335, ILE:339
3	Chitosan-Zn-Ligated Hydroxychloroquine	−9.17	GLY:13, SER:14, LEU: 17, GLU:18, TRP:21, GLY:49, GLY:50, CYS:52, VAL:53, TRY:110, MET:113, PHE:114, THR:335, ILE:339

## Data Availability

The original contributions presented in this study are included in the article.
